# Platelet Releasate and Extracorporeal Shock Wave Therapy (ESWT) for Treatment of a Partial Supraspinatus Tear in an Adolescent Baseball Player

**DOI:** 10.7759/cureus.61057

**Published:** 2024-05-25

**Authors:** Omar Alkhabbaz, Yasser Bibi, Morad Marikh, Daniel A Clearfield

**Affiliations:** 1 Texas College of Osteopathic Medicine, University of North Texas Health Science Center, Fort Worth , USA; 2 Orthopaedics, University of North Texas Health Science Center, Fort Worth, USA; 3 Microbiology, Immunology & Genetics, University of North Texas Health Science Center, Fort Worth, USA; 4 Microbiology, University of Texas at Arlington, Arlington, USA; 5 Sports Medicine, Motion is Medicine, North Richland Hills, USA

**Keywords:** less invasive, sports ultrasound, sports, specialty treatment, treatment

## Abstract

Supraspinatus tears are a common injury, particularly among athletes who engage in sports that include repetitive overhead motions, such as baseball players. Standard conservative therapies include rest and activity modification, physical therapy, non-steroidal anti-inflammatory drugs (NSAIDs), cold/heat therapy, and corticosteroid injections. Ongoing research and anecdotal evidence support using platelet-rich plasma (PRP) for supraspinatus/rotator cuff tears. Platelet releasate is obtained from PRP via the activation of platelets, subsequently releasing bioactive substances. Activation can be achieved through various methods, some of which include the addition of calcium chloride, thrombin, or exposure to low-level lasers. Platelet releasate has the potential to assist in the healing of tears by releasing growth factors that facilitate muscle and tendon repair.

This case presentation discusses the outcomes of platelet releasate paired with extracorporeal shock wave therapy (ESWT) for the treatment of a partial-thickness supraspinatus tear in an 18-year-old male baseball athlete. After exploring conservative treatment options, the patient opted for a single platelet releasate injection along with a four-part series with ESWT. Four weeks post-procedure, the patient reported a 25% improvement. He was able to fully return to play for the entire baseball season. Although the effectiveness of platelet releasate is still a topic of debate and further investigation, this case demonstrates how platelet releasate shows promising results in accelerating the treatment recovery for a partial supraspinatus tear. Further investigation and research could support the benefit of this procedure for accelerated recovery of injuries compared to PRP.

## Introduction

Rotator cuff injuries, which involve the group of muscles and tendons stabilizing the shoulder joint, are a prevalent orthopedic concern. These injuries often arise from repetitive overhead activities, traumatic events, or age-related degeneration, with the supraspinatus muscle, which is responsible for initiating the abduction of the arm, being the most frequently injured. Because it plays a crucial role in shoulder movement, the supraspinatus is particularly susceptible to strain and tears, contributing to the majority of rotator cuff injuries. The incidence of rotator cuff tears in the general population is 5% to 30%; the prevalence of the condition is about 25% in people over the age of 65 and 50% in individuals over the age of 80 [1]. A tear in the tendon can either occur completely or partially, leading to pain that can range from minimal to extreme. This pain is usually localized to the top of the outer aspect of the shoulder, with the pain occasionally radiating down the side of the arm. Pain when trying to sleep on their side is also typical [1]. These tears are commonly associated with overhead activities such as weightlifting, throwing, manual labor jobs, and trauma [1]. The diagnosis of rotator cuff pathology is predominantly conducted through MRI. However, musculoskeletal ultrasound is becoming a cost-effective alternative, providing real-time imaging of shoulder pathology [2].

Treatment options for supraspinatus tears have traditionally included conservative therapy or a surgical arthroscopic approach. Typically, for complete tears in patients under the age of 40, surgical treatment is recommended [3]. However, recent studies have provided high-level evidence showing surgical treatment for even full-thickness tears is weak and that conservative therapies should be initiated before exploring surgical options [4]. These studies concluded that any patient with asymptomatic tears should have nonoperative management, and any newly diagnosed full rotator cuff tears (RCT) should always start with physical therapy addressing both core and scapular muscle strengthening [1]. 

There has been significant evidence stating that patients with partial RCTs or tendinopathy experience significant clinical improvement in symptoms when treated with US-guided PRP injections compared to corticosteroids (CS) in the short-term three-month follow-up [5]. With that being said, there are almost no studies discussing platelet releasate (the supernatant of PRP) as a treatment option for RCTs. Therefore, this case report takes on the role of evaluating a young patient presenting with a partial-thickness supraspinatus tear and the novel approach of platelet releasate in conjunction with extracorporeal shock wave therapy (ESWT) that was taken for treatment.

## Case presentation

An 18-year-old right-hand dominant male baseball player who plays the catcher position presented to the clinic with right shoulder pain. The patient reported that he had a rotator cuff injury in his anterior shoulder area about nine months prior, which led to him sitting out of the previous season. He was able to rest it during that time, which led to him being able to continue playing before his clinical presentation. However, once he started throwing, the pain returned to its exact location. The patient described the pain as sharp, non-radiating, and only occurring during the cocking phase of the throw. It had caused him to halt any upper-body exercises, along with trouble sleeping due to pain. The patient had tried ice; however, it only seemed to provide temporary pain relief. The patient had also tried electrical stimulation but did not benefit. The patient also denied taking non-steroidal anti-inflammatory drugs (NSAIDs).

**Figure 1:**
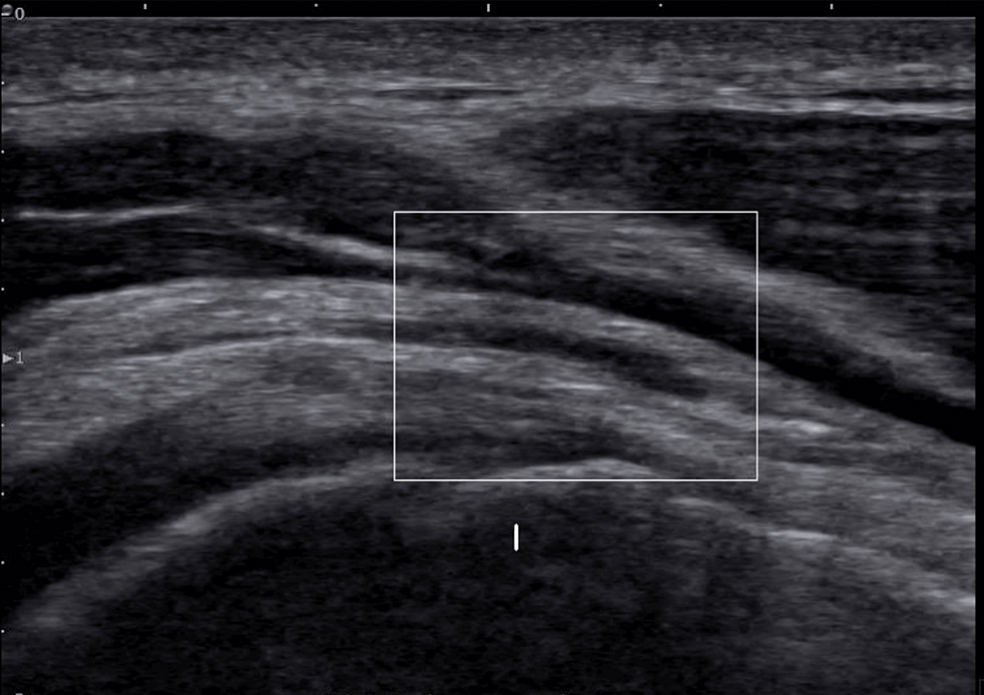
First pre-treatment ultrasound view of the right anterior supraspinatus

**Figure 2:**
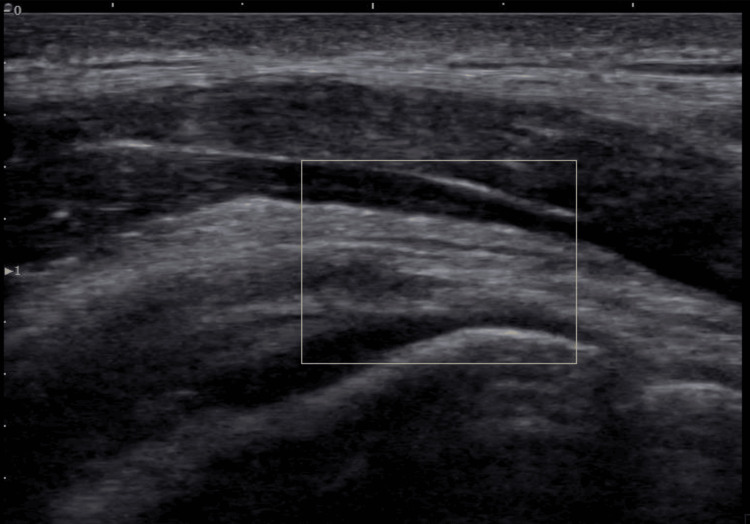
Second pre-treatment ultrasound view of the right anterior supraspinatus

On ultrasound imaging, the supraspinatus muscle showed a 25% partial tear on the bursal surface of the anterior aspect (Figures 1-2), with no retraction seen on the dynamic exam. There was no other tendinosis or tear noted, with normal muscle appearance without any atrophy or fatty infiltration or evidence of impingement with dynamic imaging. 

The nature of the injury was discussed with the patient, and the patient was given many treatment options, including physical therapy, a home exercise program, subacromial corticosteroid injections, surgical consultation, and a discussion on regenerative orthopedic medicine treatments including ESWT, platelet rich plasma (PRP), prolotherapy, and platelet releasate. The patient chose platelet releasate along with a four-part series of ESWT. 

Two days after the initial clinical visit (January 11, 2023), the procedure was conducted. The ESWT was conducted before the injection procedure. With the patient in a supine crass position, 3000 total pulses at 15 MHZ were applied to the affected area using 2.6-3.6 bars of energy with a D-Actor C15 tip. The patient exhibited good tolerance to the treatment, with a reported decrease in pain and improved range of motion. Following ESWT, a platelet releasate procedure was conducted on the right shoulder. After identifying the correct area, 90 cc of venous blood was aspirated from the antecubital fossa and spun in a Drucker 755VES centrifuge (Drucker Diagnostics, Port Matilda, PA, USA). After the collection of the PRP, Recothrom was added to the solution and spun again in the centrifuge. After the second spin, the platelet releasate was siphoned off. The area to be injected was cleaned and prepped in a sterile fashion. Topical anesthesia with Pain Ease (a skin refrigerant anesthetic spray) and a wheal of 1% lidocaine prior to injection were used. Using a 22-gauge, 2.75-inch needle, 5 mL of D25 prolotherapy was injected into the right shoulder. Then, using a separate 25-gauge, 1.5-inch needle, 8 mL of platelet releasate mixed with 0.5 cc of 0.5% ropivacaine was injected into the anterior supraspinatus and subacromial bursa under ultrasound guidance with an image capture of the procedure. The area was cleaned and bandaged after completion. There were no reportable complications. Directly after the procedure conducted on January 11, 2023, the patient reported decreased pain, increased range of motion, and good tolerance. After-care instructions were provided. Following the procedure on January 11, 2023, ESWT was conducted in the same manner on January 19, January 25, and February 9. Before conducting the last ESWT on February 9, 2023, the patient stated that he started pitching lightly one week ago, with a 25% improvement compared to baseline. He also stated that he has been experiencing new sharp pain at the posterior aspect of the rotator cuff. 

**Figure 3:**
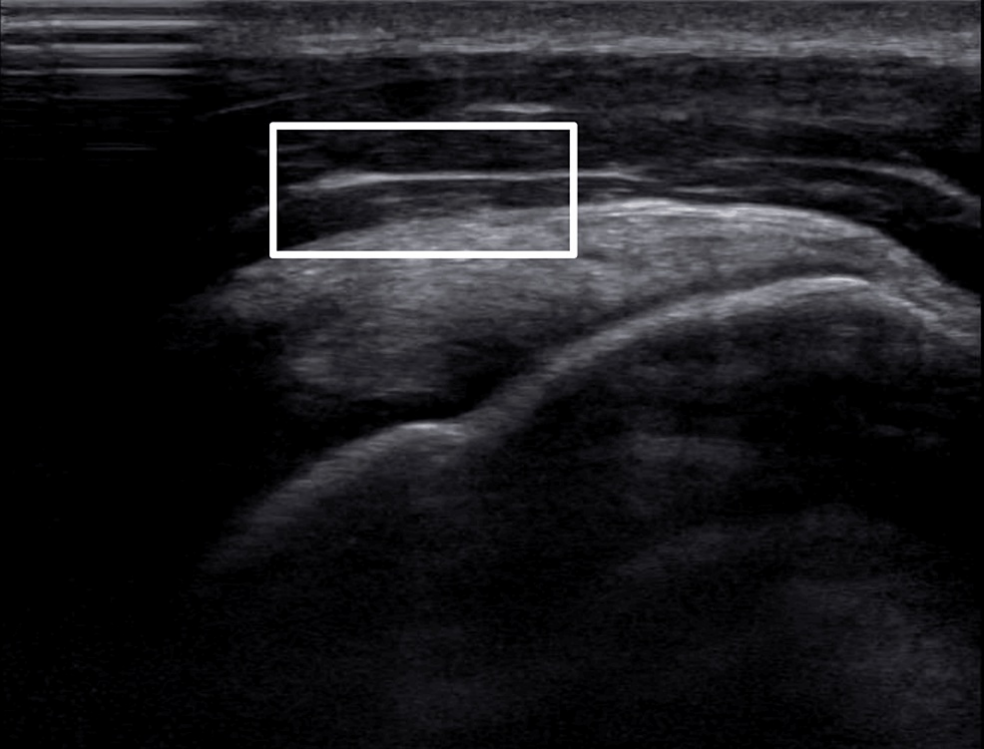
First post-treatment ultrasound view of the right anterior supraspinatus

**Figure 4:**
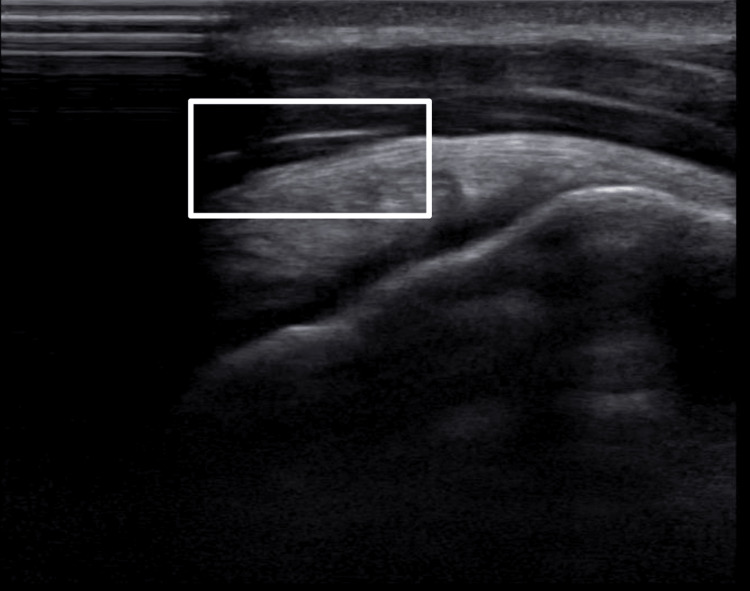
Second post-treatment ultrasound view of the right anterior supraspinatus

On imaging, the patient’s supraspinatus muscle showed significant improvement in the tear, with only 10% of the tear in the anterior supraspinatus still apparent (15% reduction total in the supraspinatus tear). There was evidence of acute supraspinatus tendinitis (Figures 3-4), with no impingement on dynamic testing. There was also some newly acquired bursitis in the subacromial bursa. There was no other noted tendinosis or tear, with a normal-looking muscle appearance. Later, after his final visit, the patient reported that his bursitis was able to calm down and that he was able to play full-time during his senior year. The patient reported returning to a high level of play after the treatment.

## Discussion

As mentioned earlier, supraspinatus (along with other muscles) tears are a common injury in sports players. Fortunately, there is a wide spectrum of treatment options to choose from; however, as we saw in this case, platelet releasate paired with ESWT seems to be a minimally-invasive, effective modality that can result in anywhere from partial to full resolution of symptoms. Although this case strongly supports the case for platelet releasate, the only arguable bias in this case is that it was paired with ESWT. Here, we review the literature on both the ESWT and platelet releasate and relate them to our case. 

Although ESWT may not be fully understood, there have been many proposed mechanisms. The first mechanism is the stimulation of mechanoreceptors, leading to a temporary reduction in pain signal transmission along with the release of endogenous opioids (natural pain-relieving substances in the body) [6]. This is consistent with our case, as our patient immediately experienced pain relief after each ESWT session. Another mechanism is ESWT stimulating the release of growth factors such as transforming growth factor-beta (TGF-β), vascular endothelial growth factor (VEGF), which stimulates angiogenesis, and platelet-derived growth factor (PDGF). These growth factors are involved in the healing process, promoting cellular proliferation, the synthesis of collagen, and the remodeling of surrounding tissue [7]. Although these independent growth factors were not measured in our case, they played a large factor considering that the supraspinatus tear went from a 25% to 10% tear in less than a month. There have been other proposed mechanisms as well, such as modulation of inflammatory responses, mechanical stimulation of cellular effects, improved neurovascularization, and improved blood flow [7]. All of these factors are potential mechanisms that led to the repair and regeneration of the partially torn supraspinatus tear.

As mentioned earlier, platelet releasate is obtained as a supernatant from a patient’s PRP, which further isolates the growth factors that initiate tissue regeneration. The PRP is obtained by blood collection (typically from a peripheral vein), anticoagulant addition, centrifugation, and PRP collection using sterile techniques [8]. Although the onset of action between PRP and platelet releasate still yields more research due to the limited literature, platelet releasate is hypothesized by physicians to have a faster onset of action compared to conventional PRP due to the activation of platelets to release their bioactive molecules and growth factors prior to injection. Some of these bioactive molecules and growth factors include PDGF, VEGF, epidermal growth factor (EGF), various cytokines, chemokines, and adhesive proteins that can collectively assist in tissue inflammation and healing [9]. These growth factors are the same ones that ESWT stimulates the release of, which supports the growth factor mechanism of rotator cuff recovery. Platelet releasate has also been reported to provide pain relief and functional improvement, with some studies showing better outcomes than corticosteroid injections (specifically for common shoulder diseases) [10]. The mechanism of this pain relief is also attributed to the mentioned growth factors and anti-inflammatory agents. This is consistent with this case, as our patient reported decreased pain every visit after his injection. Finally, one last mechanism worth mentioning regarding the healing properties of platelet releasate is the stimulation of collagen production from fibroblasts, which can promote the formation of new, stronger tendon tissue [11]. Platelet releasate contains fibroblast growth factor (FGF), which is responsible for extracellular matrix and collagen synthesis, which is important for the regeneration of tissue. Finally, this is also consistent with our case, as our patient underwent a 15% reduction in his RCT.

## Conclusions

Generally, it can be difficult to highlight specifics and attribute them to the therapeutic effects of a treatment that facilitates various regenerative and healing properties. The main point of this case is to surface a less popular therapy compared to PRP: platelet releasate/ESWT and their therapeutic effects for musculoskeletal-related injuries. Although our patient was able to attain a resolution of symptoms and go on to have a successful season, it is important to note that there is literature that goes against PRP/platelet releasate, and ESWT. Just like any medical treatment, there is going to be a patient population that responds well, moderately, or not at all. The medical interventions mentioned serve more as a tool on a toolbelt, meant for the diversification of treatment options. Platelet releasate paired with ESWT is a minimally invasive outpatient procedure and should be presented as a potential therapeutic treatment option to patients before considering invasive alternatives.
